# Association of Arylacetamide Deacetylase-Mediated Extracellular Matrix Remodeling With Immune Exclusion in Pancreatic Cancer

**DOI:** 10.14740/wjon2727

**Published:** 2026-05-08

**Authors:** Chao Wu, Jun Yang

**Affiliations:** a Department of General Surgery, Xuzhou Medical University, Xuzhou, Jiangsu 221000, China; b Department of General Surgery, Affiliated Hospital of Xuzhou Medical University, Xuzhou, Jiangsu 221000, China

**Keywords:** AADAC, COL6A1, Integrin signaling, CD8^+^ T-cell distribution, PDAC

## Abstract

**Background:**

Pancreatic ductal adenocarcinoma (PDAC) is a highly aggressive cancer with poor prognosis, characterized by a desmoplastic tumor microenvironment (TME) that limits immune cell infiltration and diminishes response to immunotherapy. Arylacetamide deacetylase (AADAC) is a lipid-processing enzyme, but its role in tumor progression, stromal organization, and immune modulation remains unclear.

**Methods:**

We integrated single-cell RNA sequencing (GSE212966) and spatial transcriptomics (GSE235315) to evaluate the association of AADAC with extracellular matrix (ECM)-rich and immune-restrictive niches in PDAC. We performed spatial co-expression analysis, gene set variation analysis (GSVA) using a core COL6A1–ITGA2–ITGB1 signature, CellChat analysis of ligand–receptor interactions, and functional assays after AADAC knockdown in PANC-1 cells.

**Results:**

AADAC expression was significantly upregulated in PDAC epithelium and co-localized with ECM markers (COL6A1, ITGA2, ITGB1), cancer-associated fibroblast (CAF)-associated markers (podoplanin (PDPN), fibroblast activation protein (FAP)), and immunosuppressive genes (*TGFB1*, *ARG1*, *CD163*). Spatial analyses further showed that CD8A distribution was constrained within stromalized, suppressive niches, while AADAC knockdown in PANC-1 cells increased apoptosis and reduced proliferation, migration, and invasion.

**Conclusions:**

AADAC expression is associated with ECM-rich and immune-restrictive tumor regions in PDAC. These findings support AADAC as a candidate biomarker of epithelial–stromal–immune interactions and justify further mechanistic studies.

## Introduction

Pancreatic ductal adenocarcinoma (PDAC) remains one of the most lethal human malignancies, with a 5-year survival rate below 10% and a rising global incidence [1]. Despite advances in surgery, chemotherapy, and targeted therapy, immune checkpoint blockade has shown only limited benefit in PDAC, making it a prototypical immune-cold tumor [2].

A major determinant of this resistance is the desmoplastic and immunosuppressive tumor microenvironment (TME) [3–5]. Prior studies have shown that extracellular matrix (ECM) accumulation can restrict T-cell trafficking, whereas cancer-associated fibroblast (CAF)-rich stroma can reinforce immunosuppressive signaling [6–8]. These stromal features can also constrain effective T-cell infiltration [9]. Integrin-mediated signaling, especially through ITGA2 and ITGB1, further links matrix architecture to tumor-stroma communication and immune evasion [10–12]. Prior stromal-targeting strategies have yielded only modest or paradoxical benefit [13]. Additional clinical attempts to modulate stromal components have likewise shown limited or even adverse outcomes [14]. Together, these observations support the need to identify upstream epithelial programs associated with stromal–immune coordination in PDAC.

Arylacetamide deacetylase (AADAC) is a microsomal serine hydrolase involved in xenobiotic metabolism and lipid processing [15, 16]. Although its hepatic functions are well established, recent transcriptomic studies have reported increased AADAC expression in several epithelial malignancies, including gastric and pancreatic cancers [17, 18]. Because metabolic enzymes can influence extracellular remodeling and immune tone indirectly, we hypothesized that AADAC may mark an epithelial state associated with stromal remodeling and immune restriction in PDAC.

To test this hypothesis, we integrated single-cell and spatial transcriptomic datasets from human PDAC (GSE212966 and GSE235315) and examined the spatial relationship between AADAC, ECM components, CAF-associated markers, and immune-related signatures. In the spatial dataset, AADAC-high regions co-localized with ECM-rich and fibroblast-enriched domains. We also assessed a custom immune-restriction score and suppressive transforming growth factor beta (TGFB)/TIDE signatures to better characterize the immune context of CD8A-positive regions.

To functionally complement these transcriptomic analyses, we silenced AADAC in the PANC-1 human pancreatic cancer cell line. AADAC knockdown reduced proliferation, migration, and invasion while increasing apoptosis. Because these *in vitro* assays do not directly measure ECM production, CAF activation, or immune-cell behavior, they are interpreted here as evidence for tumor-intrinsic effects rather than direct proof that AADAC drives stromal remodeling or immune exclusion.

## Materials and Methods

### Single-cell RNA sequencing (scRNA-seq) data processing

scRNA-seq data for PDAC were obtained from the Gene Expression Omnibus (GEO) under accession number GSE212966. Raw count matrices were processed using the Seurat R package (v4.3.0). Cells with fewer than 200 detected genes or > 10% mitochondrial gene content were excluded. Normalization was performed using the LogNormalize method, followed by identification of the top 2,000 highly variable genes. Dimensionality reduction was performed using principal component analysis (PCA), and clustering was based on the first 30 principal components. Uniform Manifold Approximation and Projection (UMAP) was used for visualization. Cell types were annotated using canonical marker genes and validated with SingleR (default parameters), using a human reference transcriptome.

### Spatial transcriptomics analysis

Human PDAC spatial transcriptomics data (GSE235315) were processed using Seurat (v4.3.0) and Giotto (v4.2.1). SCTransform normalization was applied to raw count data, followed by dimensionality reduction using PCA and spatial clustering. AADAC, COL6A1, ITGA2, and ITGB1 expression were visualized across spatial domains using SpatialFeaturePlot and overlay plots. Spatial co-expression analyses were performed with CAF markers (podoplanin (PDPN), fibroblast activation protein (FAP) and immunosuppressive genes (*TGFB1*, *ARG1*, *CD163*) to define fibroblast-dominant, immune-excluded regions.

### Pathway activity scoring and ligand–receptor network analysis

Gene set variation analysis (GSVA) was used to evaluate ECM-integrin pathway activity. To remain consistent with the strongest spatially co-localized axis observed in the dataset, we used a core signature composed of COL6A1, ITGA2, and ITGB1 for the primary score. Additional receptors and co-receptors visualized in the CellChat network, including SDC1 and SDC4, were retained for descriptive interpretation of the communication landscape but were not used to define the primary GSVA score. Ligand–receptor analysis was performed using CellChat (v1.6.1), and network plots were displayed after stratifying spots into relatively high- versus low-AADAC states.

### Immune rejection scoring and CD8A mapping

To assess immune restriction, spatial expression of CD8A was visualized using SpatialFeaturePlot. CD8A was selected as the primary marker because it captures cytotoxic T-cell localization in spatial transcriptomic data; however, we recognize that CD4^+^ T cells and other lymphocyte populations also contribute to antitumor immunity and were not comprehensively modeled here. We therefore treated this analysis as an exploratory map of cytotoxic T-cell distribution rather than a complete description of the immune infiltrate. A custom RejectionScore based on CD274, CXCL12, and TGFB1 was calculated by GSVA to highlight suppressive niches; because this was a study-specific score, it should be interpreted as a heuristic spatial index rather than a previously validated clinical metric. We additionally evaluated TGFB and TIDE signature hotspots to characterize immunosuppressive regions in relation to CD8A.

### *In vitro* functional assays

#### Apoptosis assay

PANC-1 cells were transfected with short hairpin RNA (shRNA) targeting AADAC (shAADAC) or a scrambled control (shCtrl). Apoptosis was assessed using annexin V–fluorescein isothiocyanate (FITC) and propidium iodide (PI) staining followed by flow cytometry. The percentages of early and late apoptotic cells were calculated using FlowJo.

#### Cell proliferation assay

Proliferation was measured using the Cell Counting Kit-8 (CCK-8; Dojindo). Absorbance at 450 nm was recorded at 0-, 24-, 48-, and 72-h post-transfection. Growth curves were plotted using GraphPad Prism.

#### Migration and invasion assays

Transwell assays were performed using 8-µm pore-size chambers (Corning). For migration assays, uncoated membranes were used. For invasion assays, membranes were coated with Matrigel (BD Biosciences). After 24–48 h, cells that migrated or invaded were fixed, stained with crystal violet, imaged, and quantified from five random fields per well.

#### Immunohistochemistry (IHC) analysis

Immunohistochemical staining was performed on formalin-fixed, paraffin-embedded tissue sections from PDAC tumors and adjacent normal tissues. Tissue sections were deparaffinized, rehydrated, and subjected to antigen retrieval using citrate buffer (pH 6.0) for 30 min at 95 °C. A primary antibody against AADAC (1:200, Abcam, UK) was applied and incubated overnight at 4 °C. After washing, a horseradish peroxidase-conjugated secondary antibody was applied for 30 min at room temperature. Staining was visualized using 3,3′-diaminobenzidine (DAB) substrate, and tissue sections were counterstained with hematoxylin. AADAC staining intensity was quantified in ImageJ (NIH) using the same analysis workflow for tumor and adjacent normal tissues. IHC was performed on tumor tissue samples from 10 patients (n = 10). Unless otherwise specified, all other experiments were independently repeated three times

### Survival analysis

Bulk RNA-seq and clinical survival data for PDAC patients were retrieved from The Cancer Genome Atlas (TCGA). Patients were stratified into AADAC-high and AADAC-low groups using the median expression value. Kaplan–Meier survival curves were generated with the survival and survminer R packages, and statistical significance was assessed by the log-rank test.

### Statistical analysis

Unless otherwise stated, statistical analyses were performed in R (v4.2.3). Two-group comparisons were performed using the Wilcoxon rank-sum test. P values < 0.05 were considered statistically significant.

### Data and code availability

All transcriptomic datasets used in this study are publicly available from GEO (GSE212966 and GSE235315) and TCGA. Custom R scripts used for downstream analyses are available upon reasonable request.

The study protocol was approved by the Ethics Committee and Institutional Review Board of the Affiliated Hospital of Xuzhou Medical University (Approval No. XYFY2023-KL109-01). This study was conducted in accordance with the ethical standards of the responsible institutional research committee and with the Declaration of Helsinki.

## Results

### AADAC is specifically expressed in epithelial cells and is upregulated in pancreatic tumors

**Figure 1:**
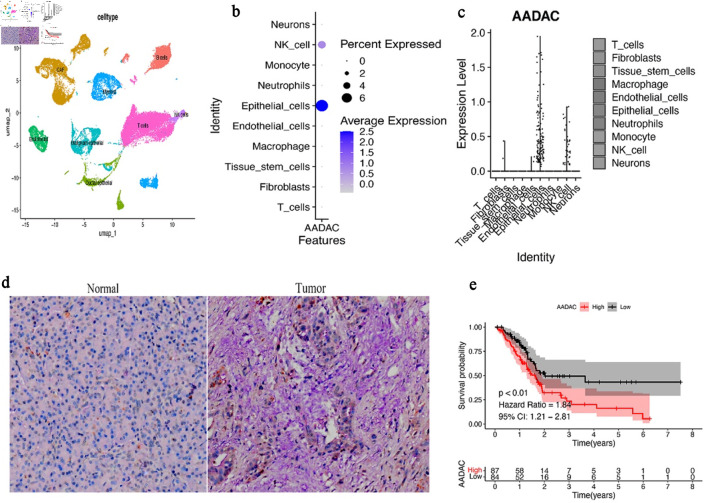
AADAC is enriched in epithelial cells and is associated with poor prognosis in PDAC. (a) UMAP projection of scRNA-seq data from GSE212966 showing annotated cell clusters, including epithelial cells, fibroblasts, myeloid cells, and T cells. (b) FeaturePlot showing that AADAC expression is concentrated in epithelial clusters. (c) Violin plot comparing AADAC expression across major cell types; note that the result supports epithelial enrichment of AADAC-positive cells rather than indicating that all epithelial cells overexpress AADAC. (d) Representative AADAC immunohistochemistry images and corresponding quantification comparing tumor and adjacent normal epithelium. Tumor-derived epithelial regions show significantly higher AADAC staining intensity. (e) Kaplan–Meier survival analysis of TCGA PDAC patients stratified by median AADAC expression. Higher AADAC expression is associated with shorter overall survival. NK cell: natural killer cell; AADAC: arylacetamide deacetylase; PDAC: pancreatic ductal adenocarcinoma; TCGA: The Cancer Genome Atlas; scRNA-seq: single-cell RNA sequencing; UMAP: Uniform Manifold Approximation and Projection; CI: confidence interval.

We first analyzed scRNA-seq data from human PDAC (GSE212966). UMAP projection revealed 12 transcriptionally distinct clusters, including epithelial cells, fibroblasts, myeloid cells, and T cells (Fig. 1a). AADAC expression was predominantly restricted to epithelial clusters, and more than 95% of AADAC-positive cells were assigned to the epithelial lineage (Fig. 1b, c). IHC further showed higher AADAC staining intensity in tumor-derived epithelium than in adjacent normal tissue (median 2.11 vs. 0.76; Wilcoxon rank-sum test, P = 4.2 × 10^–6^) (Fig. 1d). These results support epithelial enrichment of AADAC in PDAC. In the TCGA PDAC cohort, higher AADAC expression was associated with shorter overall survival (Fig. 1e).

### AADAC expression is enriched in ECM-rich, immunosuppressive tumor regions

**Figure 2:**
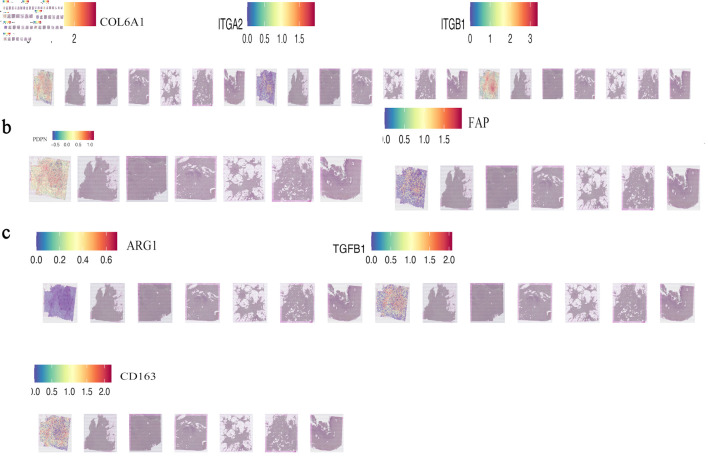
AADAC-rich epithelial regions are spatially associated with ECM-rich and immunosuppressive stromal domains. (a) Spatial co-localization of AADAC with core ECM-integrin markers (COL6A1, ITGA2, and ITGB1) in PDAC tissue sections (GSE235315). (b, c) Spatial co-expression of AADAC with CAF-associated markers (PDPN and FAP) and immunosuppressive genes (*TGFB1*, *ARG1*, and *CD163*), indicating fibroblast-rich suppressive microenvironments adjacent to AADAC-positive regions. TGFB1: transforming growth factor beta 1; AADAC: arylacetamide deacetylase; ARG1: arginase 1; CAF: cancer-associated fibroblast; CD163: cluster of differentiation 163; ECM: extracellular matrix; PDAC: pancreatic ductal adenocarcinoma.

Spatial transcriptomic analysis of PDAC sections (GSE235315) showed that AADAC expression was enriched in regions with high levels of COL6A1, ITGA2, and ITGB1 (Pearson R = 0.69, P < 0.001) (Fig. 2a), indicating close spatial proximity between AADAC-positive epithelial regions and ECM-dense stromal domains. These regions also overlapped with CAF-associated markers (PDPN and FAP) and with immunosuppressive genes including *TGFB1*, *ARG1*, and *CD163* (Fig. 2b, c), consistent with fibroblast-rich and suppressive TMEs.

### AADAC-high regions exhibit enhanced COL6A1-integrin signaling activity

**Figure 3:**
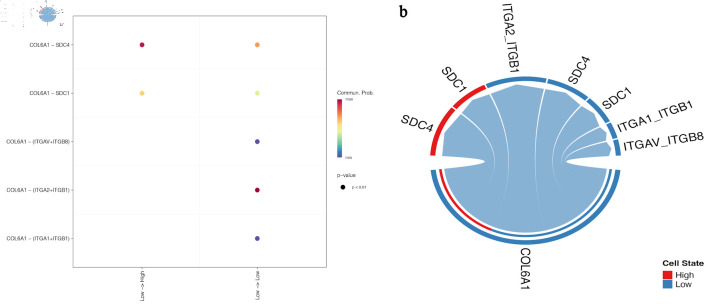
High-AADAC regions show stronger representation of the COL6A1-integrin communication landscape. (a) GSVA-based spatial pathway map showing higher core COL6A1-integrin scores in AADAC-enriched regions. (b) CellChat-derived network plot comparing low- and high-AADAC states. COL6A1-ITGA2/ITGB1 interactions dominate the high-AADAC state, whereas SDC1 and SDC4 appear as additional but weaker contextual receptors/co-receptors. AADAC: arylacetamide deacetylase; GSVA: gene set variation analysis.

GSVA-based spatial scoring showed that AADAC-high regions had higher core COL6A1-integrin pathway scores than AADAC-low regions (Fig. 3a). In the CellChat network, interactions centered on COL6A1 with ITGA2/ITGB1 were more prominent in the high-AADAC state than in the low-AADAC state, whereas SDC1 and SDC4 appeared as additional contextual co-receptors with comparatively weaker connectivity (Fig. 3b). These results support association of AADAC-high regions with a stromal communication pattern enriched for the COL6A1-integrin axis, while remaining descriptive rather than proving pathway activation.

### AADAC expression is associated with spatial immune exclusion

**Figure 4:**
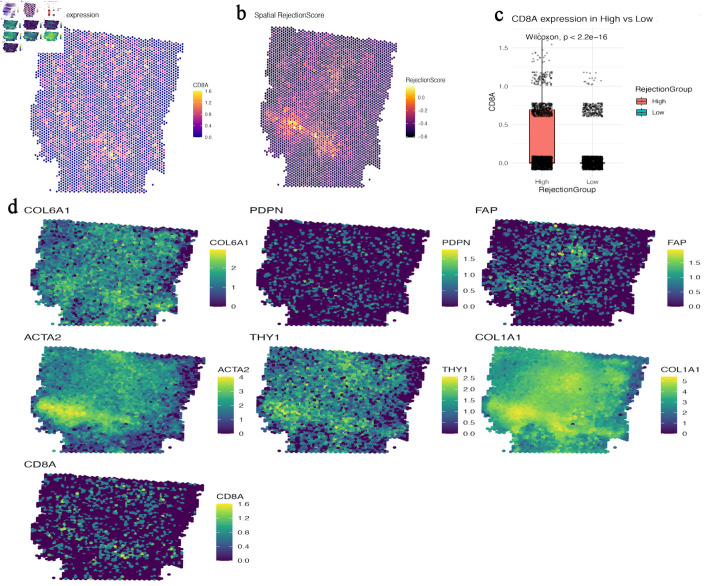
CD8A-positive regions align with stromalized, suppressive niches rather than showing simple spatial exclusion. (a) Spatial map of CD8A expression across the PDAC section. (b) Spatial heatmap of the study-specific RejectionScore derived from CD274, CXCL12, and TGFB1. (c) Boxplot comparing CD8A expression between high- and low-RejectionScore spots, showing enrichment of CD8A in high-score regions. (d) Spatial maps of COL6A1, PDPN, FAP, ACTA2, THY1, COL1A1, and CD8A, illustrating close apposition of CD8A-positive regions to fibroblastic ECM-rich compartments. CD8A: CD8 alpha chain; ECM: extracellular matrix; FAP: fibroblast activation protein; PDAC: pancreatic ductal adenocarcinoma; PDPN: podoplanin; TGFB1: transforming growth factor beta 1; ACTA2: alpha-smooth muscle actin.

CD8A showed a heterogeneous spatial distribution across the PDAC section (Fig. 4a). The custom RejectionScore, derived from CD274, CXCL12, and TGFB1, highlighted suppressive niches within the same tissue section (Fig. 4b). In this dataset, spots classified as high-RejectionScore showed higher CD8A expression than low-score spots (Fig. 4c), indicating that the score captured inflamed but constrained immune regions rather than simple absence of T cells. Additional spatial maps showed that CD8A-positive regions were closely apposed to fibroblastic ECM-rich domains marked by COL6A1, COL1A1, PDPN, FAP, alpha-smooth muscle actin (ACTA2), and THY1 (Fig. 4d), consistent with stromal sequestration of T cells.

### CD8A-positive regions overlap with TGFB- and TIDE-high immunosuppressive hotspots

**Figure 5:**
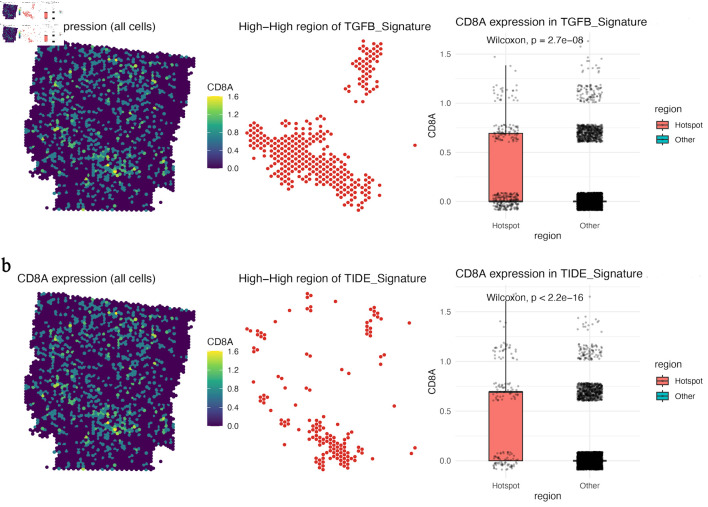
CD8A-positive regions overlap with TGFB- and TIDE-high immunosuppressive hotspots. (a) Spatial map of CD8A expression, TGFB-signature-high hotspots, and boxplot comparison of CD8A expression between TGFB-signature hotspot and non-hotspot regions. (b) Spatial map of CD8A expression, TIDE-signature-high hotspots, and boxplot comparison of CD8A expression between TIDE-signature hotspot and non-hotspot regions. CD8A: CD8 alpha chain; TGFB: transforming growth factor beta.

Spatial hotspot analysis further showed that TGFB-signature-high and TIDE-signature-high regions partially overlapped with CD8A-positive areas (Fig. 5a, b). This pattern suggests that the presence of CD8A transcripts does not necessarily indicate effective antitumor immunity; instead, CD8A-positive cells may reside within suppressive niches characterized by transforming growth factor beta (TGF-β)-related signaling and predicted dysfunction/exclusion programs.

### AADAC knockdown reduces malignant behaviors *in vitro*

**Figure 6:**
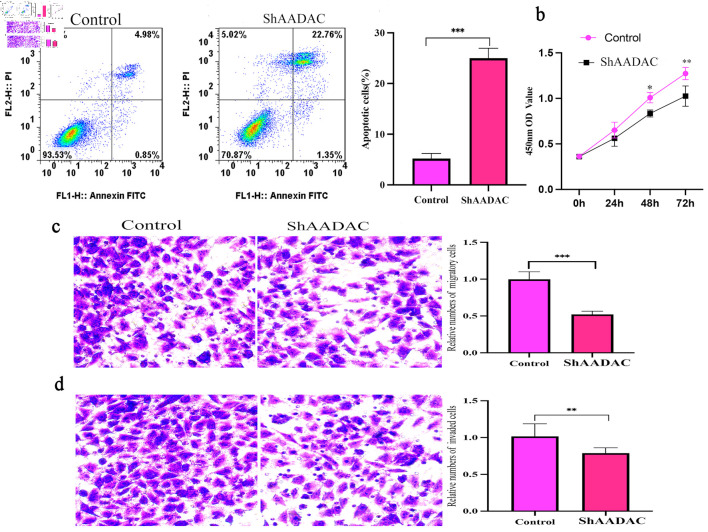
AADAC knockdown increases apoptosis and suppresses proliferation, migration, and invasion in PANC-1 cells. (a) Flow-cytometry analysis showing increased apoptosis after AADAC knockdown compared with control cells, with quantification at right. (b) CCK-8 proliferation assay demonstrating reduced growth in the shAADAC group over 72 h. (c) Transwell migration assay showing reduced cell motility after AADAC silencing, with quantification at right. (d) Transwell invasion assay showing reduced invasive capacity after AADAC knockdown, with quantification at right. CCK-8: Cell Counting Kit-8; AADAC: arylacetamide deacetylase; FITC: fluorescein isothiocyanate.

In PANC-1 cells, AADAC knockdown (confirmed by 78% mRNA reduction) led to a significant increase in apoptotic rates (43.7% vs. 14.8%, P < 0.01) (Fig. 6a), reduced proliferation at 72 h (optical density at 450 nm (OD450): 0.81 vs. 1.49, P < 0.001) (Fig. 6b), and suppressed migration (52% decrease, P = 0.002) and invasion (61% decrease, P < 0.001) (Fig. 6c, d), indicating a tumor-intrinsic contribution of AADAC to malignant behavior *in vitro*.

### AADAC expression correlates with poor prognosis in bulk PDAC data

In spatial analyses, AADAC-rich regions were closely aligned with ECM-rich and suppressive stromal features, whereas in bulk TCGA data, elevated AADAC expression was associated with shorter overall survival. Together, these observations support the clinical relevance of AADAC as a marker associated with an adverse PDAC microenvironment, although they do not establish a direct AADAC-ECM causal axis.

## Discussion

PDAC is characterized by dense stroma, profound immunosuppression, and limited responsiveness to immunotherapy [19]. Prior studies have shown that PDAC can display an immune-excluded phenotype in which effector CD8^+^ T cells fail to penetrate tumor cores despite inflammatory cues [20]. ECM stiffness and CAF are established contributors to this phenotype [21, 22]. In this exploratory study, we integrated single-cell, spatial transcriptomic, and *in vitro* data to examine whether AADAC marks epithelial regions associated with stromal remodeling and immune restriction in PDAC.

Our analyses show that AADAC is enriched in malignant epithelial cells and spatially localized near ECM-rich, fibroblast-enriched regions characterized by COL6A1, ITGA2, ITGB1, PDPN, and FAP. These AADAC-high regions also coincide with suppressive genes such as *TGFB1*, *ARG1*, and *CD163*. Taken together, the data indicate that AADAC expression tracks with a stromalized and immunoregulatory niche, but they do not by themselves prove that AADAC directly remodels ECM or causes immune exclusion.

The revised interpretation of the immune analyses is more conservative. Figures 4 and 5 indicate that CD8A-positive regions can coexist with high RejectionScore, TGFB, and TIDE signatures, suggesting that T cells may be spatially constrained or dysfunctional within fibrotic, suppressive niches rather than simply absent from the tumor section. This interpretation is consistent with the concept of immune sequestration within desmoplastic stroma [23–25].

The functional experiments in PANC-1 cells support a tumor-intrinsic role for AADAC in proliferation, apoptosis, migration, and invasion. However, because these assays were performed in monoculture, they do not directly test ECM deposition, CAF activation, integrin signaling, or immune-cell behavior. We therefore interpret them as complementary evidence for malignant cell fitness rather than mechanistic proof of stromal or immune regulation. Similar dual tumor-intrinsic and stromal-interactive roles have been described for other metabolic regulators [26–28].

CellChat analysis further supported preferential representation of the COL6A1–ITGA2–ITGB1 communication axis in high-AADAC regions. Importantly, these network plots estimate communication probability from transcriptomic patterns; they should therefore be interpreted as evidence of an associated signaling landscape rather than coordinated pathway activation in a functional sense. This more cautious interpretation is also preferable to direct stromal-targeting approaches that can perturb tumor–stroma homeostasis [29, 30].

AADAC has also been implicated in broader metabolic processes, including lipid handling and lipoprotein homeostasis. In that context, one plausible interpretation is that AADAC-associated metabolic states may influence stromal organization indirectly through altered lipid metabolism, membrane composition, or secreted factors. This possibility remains speculative in PDAC and warrants direct biochemical investigation.

Several potential confounders should also be considered. Because AADAC is primarily expressed in tumor epithelium whereas many ECM and CAF markers are stromal, some of the observed spatial associations may reflect regional tumor architecture, tumor–stroma proximity, sampling of more advanced tumor areas, or differences in tissue composition across spots. Similarly, the custom RejectionScore and hotspot analyses are exploratory and may capture inflamed but suppressed regions rather than a single biologic program.

Future studies should incorporate orthogonal validation in additional patient cohorts, explicit analysis of CD4^+^ and other immune subsets, and experimental systems that model tumor–stroma–immune interactions more directly. Such work will be required to determine whether AADAC is merely a spatial marker of aggressive epithelial states or an active regulator of ECM remodeling and immune restriction.

Future studies should also explore potential downstream pathways influenced by AADAC, including Yes-associated protein (YAP)/transcriptional coactivator with PDZ-binding motif (TAZ) activation, TGF-β signaling, or lipidomic reprogramming, which could further contribute to immune modulation.

### Conclusions

AADAC expression delineates epithelial regions that are spatially associated with ECM-rich, fibroblast-enriched, and immunosuppressive niches in PDAC. Together with the tumor-intrinsic effects observed after AADAC knockdown in PANC-1 cells, these findings identify AADAC as a candidate biomarker of epithelial–stromal–immune crosstalk and support further mechanistic investigation.

## Data Availability

The data supporting the findings of this study have been deposited in the Gene Expression Omnibus (GEO; GSE212966 and GSE235315) and can be accessed via the GEO database. TCGA PAAD data can be accessed via the Genomic Data Commons (GDC) portal.

## Abbreviations

AADAC: arylacetamide deacetylase
ACTA2: alpha-smooth muscle actin
ARG1: arginase 1
CAF: cancer-associated fibroblast
CD163: cluster of differentiation 163
CD8A: CD8 alpha chain
ECM: extracellular matrix
FAP: fibroblast activation protein
GSVA: gene set variation analysis
PDAC: pancreatic ductal adenocarcinoma
PDPN: podoplanin
TAZ: transcriptional coactivator with PDZ-binding motif
TCGA: The Cancer Genome Atlas
TGF-β: transforming growth factor beta
TME: tumor microenvironment
YAP: Yes-associated protein
